# Accused of Witchcraft and Burned to Death

**Published:** 1888-10

**Authors:** 


### ACCUSED OF WITCHCRAFT AND BURNED TO DEATH.

An account of the burning of a young squaw by Mojave Indians, because she was accused of preaching witchcraft, comes from Los Angeles. The scene of the torture was near the Colorado River in the eastern end of San Bernardino county. For the past two months a strange disease has attacked members of the tribe, who live in a state of semi-barbarism. The victims were seized with violent headaches, purging and a gradual stiffening of the limbs. In many cases the sufferers showed all the symptoms of poisoning. Death usually followed within twelve hours from the time of the attack. It was said that at least, twenty of the most popular men in the tribe have died from this mysterious disease.

The medicine men of the tribe belabored the patients with sticks in order to drive the evil spirit out of the burning body, and in the case of Stone Head, a sub-chief, the doctors dragged the patient through the waters of the Colorado to cleanse him of the evil spirit, which, it was supposed, had taken possession of him. The spread of the disease at last became so alarming that the members of the tribe became panic stricken and slaughtered their dogs and burros as a sacrifice to appease the anger of the Great Father.

This proving of no avail, a council was held while the moon was in its last quarter. Every buck in the tribe was present. The medicine men sat around a huge pot, which was filled with herbs, while the bucks were squatted in a semi-circle some distance away. The medicine men watched the steaming of the herbs until the mess had been boiled down to a teacupful of liquid. Then a male pigeon and his mate were taken from a wicker basket and held by the medicine men while the liquid from the herbs was poured down their throats. The male bird, when released, flew away and was not seen again. The female bird fluttered a few yards and then fell, helpless and dying, to the ground. The medicine men now seemed crazed with excitement. They leaped to their feet and danced demoniacally, while the bucks sat in sullen silence.

While the medicine men were in the midst of their orgies they declared that there was a witch in the tribe. The female bird had died, while the male bird had flown away into the night. This test determined the sex of the witch.

When the bucks heard these words of the medicine men they became wild with rage. Each brave suspected the other of harboring the witch in his teepee or hut, but a final test was to be made. With yells and imprecations the frenzied reds drove their women to the place where the council had been held, and where the white pigeon still lay among the herbs and grass. The squaws were driven past in single file. The medicine men watching the face of each as she passed the bird, whose plumage looked like snow in the bright moonlight. Nearly all the women passed the pigeon without giving it any notice. Finally a young squaw, the daughter of Cresco, also a sub-chief, stepped out of the ranks and was about to pick up the bird when the medicine men, with loud yells, seized the girl and pinioned her arms. The unfortunate squaw pleaded piteously for her life, which she seemed to forsee was in peril, but her cries were of no avail, her own relatives assisting in dragging her to the council place. The death of the female pigeon was conclusive evidence that a squaw was the witch. The first squaw to touch the bird was the fatal test of guilt.

The poor girl, who was only eighteen years old, was stripped of her clothing, tied to a stake, and a slow fire built under her. For two hours she lingered in awful agony, and while her death screams filled the air the braves danced about the fire and the medicine men performed incantations. When morning came nothing but the whitened bones of the girl and the black embers of the fire remained about the stake. The disease from which so many of the Mojave braves have died, is believed to be malignant typhoid fever. The details of this strange story were brought to Los Angeles by ranchers who had been attracted to the camp of the Indians by the noises which attended the terrible death of the girl.

